# Supplementary material for: Elevated estradiol levels on hCG trigger day adversely effects on the clinical pregnancy rates of blastocyst embryo transfer but not cleavage-stage embryo transfer in fresh cycles: a retrospective cohort study
Source: PeerJ. 2023 Jul 18;11:e15709. doi: 10.7717/peerj.15709 (PMC10361074; doi:10.7717/peerj.15709)
Supplement: Supplemental Information 3 [file peerj-11-15709-s003.docx]

# Codebook for analysis

**Data names**

Blastocyst_stage_data.xlsx

Clevage_stage_data.xlsx

**Data description:**

The dataset includes information on the following variables:

- **Date of oocyte retrieval**: date-type variable (year/month/day or year-month-day).
- **Previous cycle**：The times of cycles the patient received before current cycles (0; 1-2; ≥ 3 in times).
- **female age**: The age of the subject, measured in years.
- **COH (Controlled ovarian hyperstimulation) protocol**: The COH regimen received by the patient. (GnRH-antagonist; GnRH-agonist long protocol).
- **BMI**: The body mass index of the patient, measured in kg/m^2^.
- **Sterility classification**: Type of patients’ infertility (primary; secondary)
- **infertility indication**: The cause of the patient's infertility. (DOR (decreased ovary reserve); Endometriosis; male factor; Mixed; Ovulatory; Tubal; Unexplained)
- **Duration of infertility**: measured in years
- **Fertilization type**: The fertilization technique the patient received. (IVF; ICSI)
- **basal FSH**: Basal follicle stimulating hormone in patients, measured in mIU/ml.
- **basal LH:** Basal luteinizing hormone in patients, measured in mIU/ml.
- **basal E_2_**: Basal estradiol levels in patients, measured in pg/ml.
- **hcg day FSH**: Follicle stimulating hormone on trigger day in patients, measured in mIU/ml.
- **E_2_ set**: Patients were grouped according to E2 levels on trigger day. Set 1 (E_2_ ≤ 2000 pg/ml); Set 2 (E_2_ = 2001-3000 pg/ml); Set 3 (E_2_ = 3001-4000 pg/ml); Set 4 (E_2_ > 4000 pg/ml).
- **hcg day E_2_**: Estradiol levels on trigger day in patients, measured in pg/ml.
- **hcg day LH**: Luteinizing hormone on trigger day in patients, measured in mIU/ml.
- **hcg day P**: Progesterone levels on trigger day in patients, measured in pg/ml.
- **endometrial thickness**: Endometrial thickness on trigger day, measured in mm.
- **No. of retrieved oocytes**: The number of retrieved oocytes.
- **No. of inviable embryos**: The number of inviable embryos
- **date of embryo transfer**: date-type variable (year/month/day or year-month-day)
- **No. of embryos transferred**: The number of embryos transferred.
- **No. of high-quality embryos transferred**: The number of high-quality embryos transferred.
- **OHSS**: Ovarian hyperstimulation syndrome (0-No; 1-Yes).
- **Clinical pregnancy**: Whether the patient meets the diagnosis of clinical pregnancy (0-No; 1-Yes)
- **Pregnancy classification**: Type of clinical pregnancy. (Ectopic pregnancy; heterotopic pregnancy; intrauterine pregnancy)
- **Miscarriage**: Whether the patient meets the diagnosis of clinical pregnancy (0-No; 1-Yes)
- **data of childbirth**: date-type variable (year/month/day or year-month-day)
- **Gestational age**: Patient's gestational age at delivery, measured in weeks.
- **delivery method**: Type of delivery. (Cesarean; vaginal)
- **baby 1 gender**: The sex of first child (male; female).
- **baby 1 birth weight:** The neonatal weight of first child, measured in kg.
- **baby 2 gender**: The sex of second child (male; female).
- **baby 2 birth weight**: The neonatal weight of second child, measured in kg.

**codebook in R:**

- **Generalized additive model (GAM) analysis**

This R code reads data from the clipboard and fits two binary logistic regression models using the **glm()** function from the stats package. The first model (model1) predicts the occurrence of clinical pregnancy using single predictor variable described in manuscript. The second model (model2) predicts the occurrence of the clinical pregnancy using multiple predictor variables Age (smooth), BMI, infertility duration, sterility classification, previous cycle attempts, basal FSH, basal LH, basal E2, FSH on hcg day, LH on hCG day, P on hCG day, endometrial thickness on hCG day, number of retrieved oocytes, the number of inviable embryos, and the number of embryos transferred. The **broom package** is used to extract model coefficients, confidence intervals, and p-values, which are then combined into a data frame for each model.

Specifically, the code includes the following steps:

Data loading: Data is read from the clipboard and stored in a variable called mydata using the read.delim() function.

Model 1: A univariates logistic regression model is fit using glm(). The response variable is "clinical pregnancy" and the predictor variable is "female age"(or any other vairables). The model is stored in a variable called model1.

Broom package installation and loading: The broom package is installed using the install.packages() function and loaded into the R session using the library() function.

Tidy model 1 output: The tidy() function from the broom package is used to extract model coefficients, standard errors, confidence intervals, and p-values for model1. The results are stored in a data frame called y.

Model 1 results combination: The results from model1 are combined into a data frame called z. The data frame includes the exponentiated coefficients, exponentiated confidence intervals, and p-values for the predictors in model1.

Model 2: Another logistic regression model is fit using glm(). The response variable is "clinical pregnancy" and the predictor variables are Age (smooth), BMI, infertility duration, sterility classification, previous cycle attempts, basal FSH, basal LH, basal E2, FSH on hcg day, LH on hCG day, P on hCG day, endometrial thickness on hCG day, number of retrieved oocytes, the number of inviable embryos, and the number of embryos transferred. The model is stored in a variable called model2.

Tidy model 2 output: The tidy() function from the broom package is used to extract model coefficients, standard errors, confidence intervals, and p-values for model2. The results are stored in a data frame called m.

Model 2 results combination: The results from model2 are combined into a data frame called n. The data frame includes the exponentiated coefficients, exponentiated confidence intervals, and p-values for the predictors in model2.

- **Generalized additive model (GAM) analysis**

Libraries:

The following libraries are required to run the R code: **mgcv, writexl, dplyr.**

Model Specification:

A GAM model is specified using the mgcv library with the formula:

Clinical pregnancy ~ s(hcg day E_2_, fx = FALSE) + Age (smooth), BMI, infertility duration, sterility classification, previous cycle attempts, basal FSH, basal LH, basal E2, FSH on hcg day, LH on hCG day, P on hCG day, endometrial thickness on hCG day, number of retrieved oocytes, the number of inviable embryos, and the number of embryos transferred

Model Fitting:

The GAM model is fit using the predict.gam function with the argument type = "terms" to get the smoothed function of hcg day E_2_.

The model fit and standard errors are then added to the original dataset using the cbind function.

Model Validation:

To check the model fit, a plot is created with the smoothed function of hcg day E_2_ and the upper and lower limits of the 95% confidence interval.

An algorithm is then used to identify the cutoff point of hcg day E_2_ based on the minimum point of the AIC value. A new dataset is created with hcg day E_2_, the cutoff point, and the difference between hcg day E_2_ and the cutoff point.

Three models are then fitted to the new dataset to assess the effect of the cutoff point on the relationship between hcg day E_2_ and clinical pregnancy.

Results:

The following results are generated from the R code:

The cutoff point of hcg day E_2_ is printed to the console.

The summary statistics of the three models are printed to the console.

The likelihood ratio test p-value for the significance of the cutoff point is printed to the console.
